# Analysis of Genetic and Non-Genetic Factors Influencing Timing and Time Perception

**DOI:** 10.1371/journal.pone.0143873

**Published:** 2015-12-07

**Authors:** Alex J. Bartholomew, Warren H. Meck, Elizabeth T. Cirulli

**Affiliations:** 1 Center for Applied Genomics and Precision Medicine, Duke University School of Medicine, Durham, NC, 27708, United States of America; 2 Department of Psychology and Neuroscience, Duke University, Durham, NC, 27708, United States of America; CEA.DSV.I2BM.NeuroSpin, FRANCE

## Abstract

Performance on different psychophysical tasks measuring the sense of time indicates a large amount of individual variation in the accuracy and precision of timing in the hundredths of milliseconds-to-minutes range. Quantifying factors with an influence on timing is essential to isolating a biological (genetic) contribution to the perception and estimation of time. In the largest timing study to date, 647 participants completed a duration-discrimination task in the sub-second range and a time-production task in the supra-second range. We confirm the stability of a participant’s time sense across multiple sessions and substantiate a modest sex difference on time production. Moreover, we demonstrate a strong correlation between performance on a standardized cognitive battery and performance in both duration-discrimination and time-production tasks; we further show that performance is uncorrelated with age after controlling for general intelligence. Additionally, we find an effect of ethnicity on time sense, with African Americans and possibly Hispanics in our cohort differing in accuracy and precision from other ethnic groups. Finally, a preliminary genome-wide association and exome chip study was performed on 148 of the participants, ruling out the possibility for a single common variant or groups of low-frequency coding variants within a single gene to explain more than ~18% of the variation in the sense of time.

## Introduction

The perception and estimation of time, both on a behavioral and neuroanatomical level, remains a heavily researched and theorized topic [1]. A central divide in the models used to account for timing in the hundredths of milliseconds-to-minutes range is over the presence of a centralized, general purpose clock versus localized, sensory specific mechanisms that reflect time-dependent changes in the state of neural networks [2–5]. Dedicated-clock theories of timing are typically described in terms of information-processing (IP) models that utilize a pacemaker-accumulator system [6, 7] or coincidence-detection models that utilize the synchronization of oscillatory processes [4, 8]. In the typical IP model, a Poisson pacemaker emits pulses at a high rate that pass through a mode-control switch into an accumulator. The clock reading at any moment in time is equivalent to the magnitude of pulse accumulation, which is linearly related to the passage of time [9]. Attentional time-sharing constructs can be used to extend these models by emphasizing the effect of resource allocation on pulse accumulation, in addition to the biological factors inherent to the pacemaker-counter model [10–12]. While most of these models were developed for tasks spanning multiple second intervals, they are now being extended into shorter time ranges [8, 13–15].

Multiple time-scale proponents have suggested that distinct timing mechanisms operate at varying duration ranges (e.g., micro—milliseconds, seconds—minutes, hours—days) and modes of presentation (e.g., trial number, explicit vs. implicit), possibly with varying precision [16–19]. Researchers look for transitions or ‘break points’ where one timing mechanism stops operating and/or loses sensitivity and another takes over. This is indicated by sudden jumps in the sensitivity to time as measured by the coefficient of variation (CV), which is the standard deviation of the time estimations or productions divided by the mean duration(s). The scalar property of timing implies that the CV should remain constant, but recent work highlights an inconsistency in a small range from 1 to 1.9 seconds [20]. Additional support for multiple mechanisms stems from pharmacological studies in humans where duration discrimination performance is differentially affected in the millisecond or second range [21]. Further, neuroimaging studies have identified differential activation for sub- and supra-second intervals during temporal discrimination tasks [13, 14]. Despite these lines of evidence, a longitudinal study examining the CV of human participants across a broad range of intervals, from milliseconds to minutes long, failed to find clear and consistent break points, suggesting that the transition from one type of timing mechanism to another may be more cooperative and gradual than initially thought [22, 23].

In clock-based models, the pulse emission or oscillation rate forms the basic unit of time [24]. Estimations of time, such as that performed during a prospective time production task, rely on this internal clock to produce or detect a specified target duration. When requesting the production of the same target duration from different participants, some responders will overproduce while others will underproduce the duration in objective time, but each individual experiences the same subjective duration (e.g., pulse accumulation or oscillatory pattern) due to a differing pace of the internal clock [25]. This rate remains stable within an individual but varies widely in the general population [26, 27]. Time production tasks can serve as effective tools in evaluating these individual differences in clock speed and/or the storage of the clock reading into memory [28, 29]. The rate of functioning of the internal clock may be associated with one’s preferred spontaneous motor tempo and may also be modulated by working memory resources [8, 22, 30, 31]. The pulse emission rate can be manipulated by a number of factors, including resource allocation, pharmacological agents, brain lesions, and click training [8, 32, 33]. Further, subjective time perception may be influenced by factors like gender, age, psychiatric and neurological disorders, and hormonal and circadian rhythms [8, 34–37]. Understanding and characterizing these factors is essential to isolating biological implications, such as the role of genetics, in time perception [25]. It should be noted, however, that the current study uses a duration discrimination procedure in the sub-second range and a temporal production procedure in the supra-seconds range, thereby suggesting caution when making comparisons of accuracy and precision across these tasks.

A host of neurological and psychiatric disorders sharing a connection to dopaminergic pathways, such as Parkinson’s disease, depression, schizophrenia, and attention deficit/hyperactivity disorders, have documented distortions in time perception [38–42]. Dopamine plays an important role in cognitive processes, and its effect on time perception is well established in both human and animal models [10]. Recent work suggests that individual variation in sense of time could be due to sensitivity to dopaminergic systems [33]. The impact of genotypes shown to affect striatal dopamine and related receptors have been preliminarily examined in small populations with candidate gene studies, but more powerful studies that also take into account confounding factors are necessary [25, 43].

Genome-wide association studies (GWAS) are a relatively new way for researchers to identify genes involved in complex human traits such as timing and time perception. This method searches the genome for small variations whose genotypes are correlated with a trait of interest. Each study examines hundreds of thousands of variants that are chosen to represent essentially all of the common (minor allele frequency >~5%) genetic variation in the genome. Researchers use data from this type of study to pinpoint common genetic variants that may contribute, for example, to a person’s behavioral or cognitive performance. As GWAS examine variants across the genome, they present an unbiased approach to identify common variation that contributes to complex traits. Although this is the first report of a GWAS for timing and time perception, the expectation is that future, larger genetic studies will identify genetic variants associated with complex traits relevant to interval timing, as well as variations that affect the response of a person’s sense of time to certain drugs and influence interactions between a person’s genes and temporal properties of the environment [44, 45].

To fully characterize factors contributing to individual variability in internal clock speed, we administered 647 healthy controls a prospective time production task and a duration discrimination task. In the largest study of time sense to date, we systematically assess the effects of basic demographics, cognitive performance, circadian rhythms, depression, and ADHD status on the accuracy and precision of interval timing. In addition, we demonstrate the stability of our time production and duration discrimination tasks by having a sub-set of participants complete the measures on separate occasions, approximately 2.5 months apart. Finally, we approach the breadth of individual variation from a genetic perspective with a GWAS and exome chip study, with sub-analyses focused on previously identified candidate genes [43, 46, 47].

## Materials and Methods

### Participants and Ethics

The Duke University Institutional Review Board approved all procedures, and participants provided written, informed consent (IRB #: Pro00006828).

A total of 647 healthy participants, ranging in age from 18 to 67 years, completed the time production and duration discrimination task as part of a larger battery in the Duke Genetics of Cognition and Other Normal Variation study [48–50]. None of the 647 participants were taking a drug or combination of drugs that was decided by a pharmacist as likely to impact their cognition or be indicative of a cognitive impairment, were diagnosed with a serious neurological disorder, had a head injury resulting in memory problems, had a learning disability, or had a serious psychiatric history. A more comprehensive description of the participants can be seen in Table 1.

**Table 1:** Participant Demographics.

Variable	Mean (SD) or Count (%)
Age in years	24.90 (8.08)
Ancestry	
European	301 (46.5%)
African	97 (15.0%)
East Asian	97 (15.0%)
South Asian	52 (8.0%)
Hispanic	24 (3.7%)
Other	76 (11.8%)
Sex	
Male	255 (39.4%)
Female	392 (60.6%)
Education	
Years of education	15.23 (1.97)
Current student	455 (70.3%)
CIRENS	0.47 (1.19)
BDI	34 (5.3%)
AD[H]D	16 (2.5%)
EPQ-BV	
Extraversion	39.01 (8.27)
Neuroticism	26.85 (8.04)
Musical Training	
Formal training	354 (54.7%)
Before age of six	118 (18.2%)

Standard deviation (SD), Circadian Energy Scale (CIRENS), Beck Depression Inventory (BDI), Attention Deficit [Hyperactivity] Disorder, (AD[H]D), Eysenck Personality Questionnaire—Brief Version (EPQ-BV).

### Questionnaire

Prior to psychometric testing, the participants completed an extensive survey that queried demographics, medical history, and several standardized scales [51].

#### Circadian rhythms

The Circadian Energy Scale (CIRENS) is a two-question chronotype measure based on self-report energy levels throughout the day: once at night and once in the morning. Energy levels are described on a Likert scale: [very low (1), low (2), moderate (3), high (4), or very high (5)]. The difference between the evening score and morning score determines the overall chronotype score, ranging from -4 (most marked morning preference) to +4 (most marked evening preference) [52].

#### Depression

A total of 530 participants completed the Beck Depression Inventory-II (BDI) [53]. In accordance with guidelines, participants scoring 14 or higher are categorized as having mild depression. Participants with scores above this threshold were categorized as depressed.

#### Attention Deficit (Hyperactivity) Disorder

Participants self-reported whether or not they had ever received a formal diagnosis of Attention Deficit Disorder, with or without hyperactivity.

#### Extraversion and neuroticism

The Eysenck Personality Questionnaire, Brief Version was employed and includes two scales of 12 questions for both extraversion and neuroticism [54]. Question 24 was imputed for 83 participants that did not have a recorded response using the other eleven measures of the extraversion subscale as well as age, performance on cognitive testing, and BDI-II score with the missing value analysis function in SPSS, using expectation maximization algorithms.

#### Musical training

All participants indicated whether or not they had ever received formal musical training and whether or not that training had commenced before the age of six years old, as this has been shown to be a critical time period in the development of advanced musical skills like absolute pitch [55].

### Cognitive test

All participants took a brief battery of eleven standardized, well-known cognitive tests assessing diverse areas of cognition represented in Table 1 [48]. Principal component analysis was performed on the individual test scores to determine an overall measure of performance [48]. The first principal component (PC1) explained 41.5% of the total variation in test scores and received approximately equal loadings from all tests (Table 2). It was therefore taken as a measure of overall cognitive performance on the battery and can be considered a proxy for general intelligence.

**Table 2:** Associations with time production and duration discrimination.

Variable	Production [Counting]		Production [Timing]		Duration Discrimination	
	Uni	Multi	Uni	Multi	Uni	Multi
Intelligence	**< 0.001**; 0.018	*	**< 0.001**; 0.017	*	***<* 0.001**; -2.00	*
Ancestry		*		*		*
African	***<* 0.001**; -0.120		**< 0.001**; -0.125		**< 0.001**; 10.05	
East Asian	0.973; < -0.001		0.878; 0.003		0.921; -0.155	
South Asian	0.083; -0.031		0.535; 0.017		0.467; 1.50	
Hispanic	**0.008**; -0.066		0.162; -0.053		**< 0.001**; 10.58	
Other	0.193; -0.020		0.328; -0.022		0.224; 2.10	
Male	0.038; 0.021	0.014; 0.023	**< 0.001**; 0.061	*	NS	NS
Age	0.011; -0.002	NS	NS	NS	NS	NS
Education						
Years of education	**0.002**; 0.008	NS	0.145; 0.006	NS	**< 0.001**; -1.07	0.008; -0.760
Current student	**< 0.001**; 0.059	0.018; 0.027	0.002; 0.052	NS	**0.007**; -3.36	NS
CIRENS	0.011; 0.010	0.031; 0.008	NS	NS	NS	NS
Depression ^ǂ^	NS	0.043; 0.049	NS	0.033; 0.083	**0.017**; 7.01	NS
Musical Training ^#^						
Formal training	**0.001**; 0.036	NS	0.030; 0.035	NS	NS	NS
Before age of six	0.492; 0.010	NS	0.195; 0.027	NS	NS	NS
AD[H]D	NS	NS	NS	NS	NS	NS
EPQ-BV						
Extraversion	NS	NS	NS	NS	NS	NS
Neuroticism	NS	NS	NS	NS	NS	NS

Results of univariate (column 1) and multivariate (column 2) regressions presented as *p* value; beta coefficient. Bolded *p* values are < 0.01. NS indicates p > 0.05.

^#^ Not included in multivariate stepwise regressions because missing from 118 participants and shown not to contribute in the other 529

* Included as a covariate in all multivariate regressions due to significant association in stepwise regression.

^ǂ^ Excluded from multivariate stepwise regressions due to unavailable scores for 118 participants. Depression status was not significant in any of the three timing models when restricting to the remaining 530 participants with BDI scores.

### Time Production

The time-production task was programmed and presented to participants using MATLAB (2012a) with the Psychophysics Toolbox extension [56]. The function is millisecond accurate, but keyboard sampling rates account for an error of up to 100 msec. We considered this error to be acceptable given the target lengths of time considered for this task. Participants were guided through the task with instructions presented on the screen.

Participants viewed the text: “Hold down the space bar for XX seconds”, where XX varied based on the interval requested. When the participant depressed the space bar, the screen turned blue, indicating that the timer was active. Two practice intervals, for 4 and 8 sec, were performed without feedback and for the purpose of accustoming the participant to the required motor movements. Participants completed a single trial for 1, 3, 6, 12, and 15-sec time trials with differing instructions for Part 1 (*counting*) and Part 2 (*timing*). During the counting condition, participants were instructed to use their preferred sub-interval time keeping method and encouraged to be as accurate as possible. In the timing condition, participants were explicitly instructed to resist utilizing counting methods, including rhythmic strategies. At the end of the timing task, participants indicated whether or not they were able to resist using a counting strategy. No feedback concerning the accuracy of their timed responses was given to the participant at any time. Participants were not presented with a concurrent task as dividing attentional resources has shown to decrease time production accuracy [57]. Task type (*counting* vs *timing*) was not counterbalanced, as performance was previously shown to be unaffected based on task order [58, 59].

### Duration Discrimination

Participants completed an auditory, duration-discrimination task using a classic adaptive procedure [60, 61] implemented by the MLP MATLAB toolbox [62]. In each trial, randomized 1kHz pure tones with raised cosine onset and offset gates of 10-msec were presented in a three-alternate forced choice task at 75 dBA. Participants judged which of the three tones was longest in duration and immediate feedback was given in the form of “correct” or “incorrect”. A standard 250-msec pure tone was used as the baseline and the duration of the remaining tones was determined in real time based on participant responses and presented with 500-msec silent intervals. The maximum likelihood procedure tracked the 79% of the participant’s psychometric function, and an independent threshold was independently generated three times using 30 trials. No training trials were performed. Participants were required to pass a hearing test with no more than 10 dBA of hearing loss to be eligible [62]. This paradigm was chosen because it is typically easier to discriminate differences in time with auditory rather than visual events [8].

### Repeat Sessions

To evaluate the reliability of our tasks, a total of 46 participants completed the time production and duration discrimination tasks twice. The mean amount of time between testing sessions was 79 days (SD = 39).

### Data Analyses

#### Phenotypes

**Fig 1:**
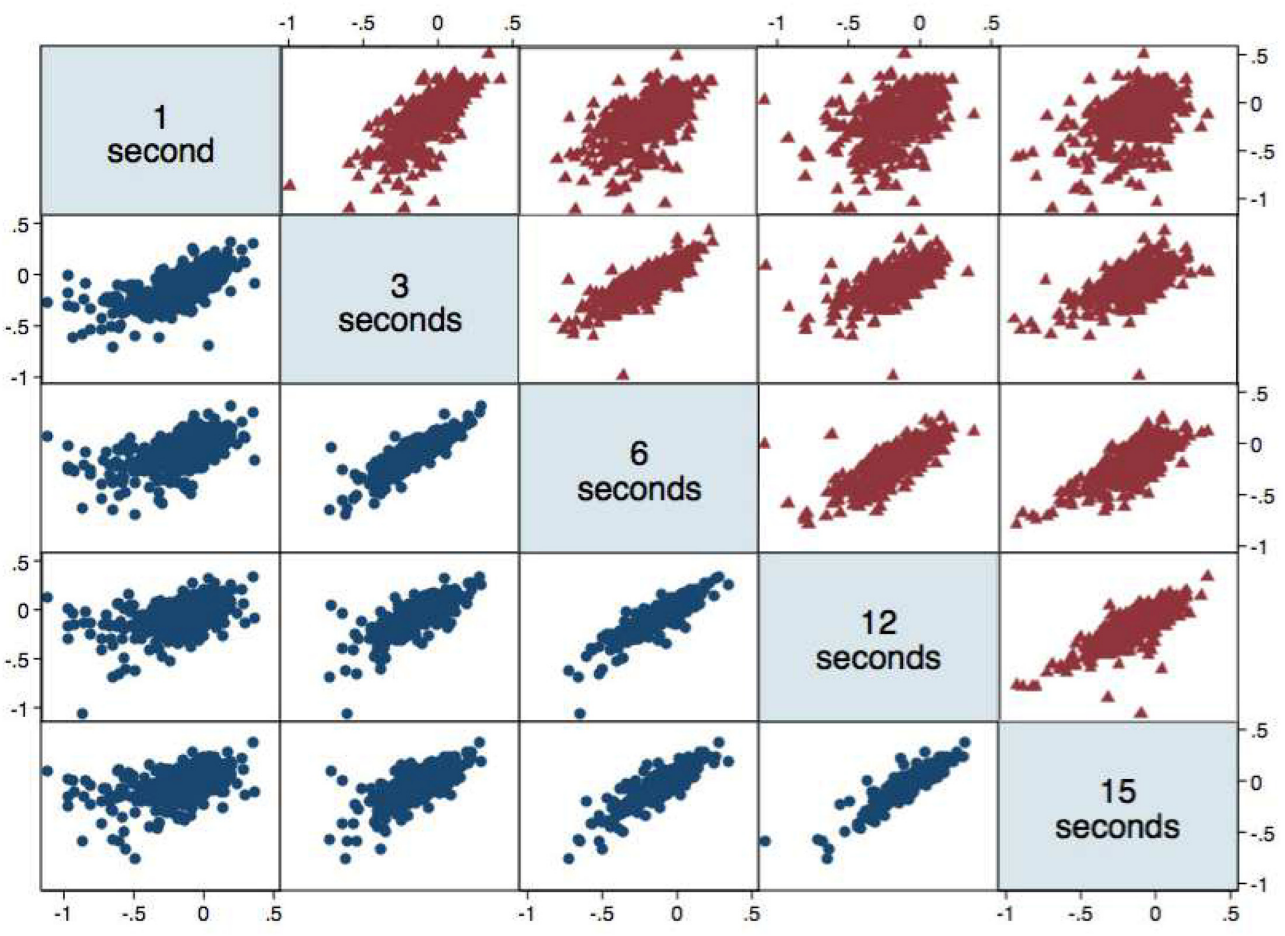
Correlations between 1, 3, 6, 12, and 15-second time production intervals. Counting (circles) and timing production (triangles) correlations are shown at each requested interval. Values are plotted as the log transformed ratio of produced time to requested time. Due to the lower correlation for the 1-second intervals, this interval was excluded from production analyses. This figure exclude individuals (n = 10) who were outliers as explained in the methods.

All statistical analyses were performed using STATA 13.1 [63]. At each requested time value in the production task, a log-transformed ratio of time produced to time requested was generated. The median of four measures, excluding the 1-sec trial, was taken as a stand-alone phenotype for participants for each condition as illustrated in Fig 1. For the discrimination task, the phenotype was the median of the three independent duration thresholds output by the maximum likelihood procedure. For all analyses, participants were excluded if their score was beyond five standard deviations of the group mean or if their individual standard deviation among trials was three times more than the group standard deviation. For the genetic analyses with duration discrimination, the aforementioned exclusionary criteria applied, in addition to excluding two individuals driving inflated association statistics due to being outliers in the more limited genetic population analysis. In addition, we restricted *timing* production to only those individuals who indicated they were able to refrain from counting at the end of the experiment. Final sample sizes for non-genetic analyses were 637 participants for *counting* production, 491 participants for *timing* production, and 629 participants for duration discrimination. For the production task, a group CV was computed at each requested interval based on group SD/mean. For the discrimination task, an individual CV is calculated based on the mean and SD of three independent thresholds.

#### Regression analyses

Stepwise forward linear regression analyses with a cutoff for inclusion of *p* < 0.01 were performed for each of the three phenotypes (*counting* production, *timing* production, and duration discrimination), with the phenotype as the outcome and all variables listed in Table 2 as covariates. Variables that were significant in the stepwise model were used as covariates in subsequent genetic analyses. The residuals of each linear analysis approximated a normal distribution.

For 118 participants, BDI -II scores were unavailable. We therefore ran the stepwise regression analysis without depression status as the final multivariate models showed no significant association with this trait.

A linear regression analysis was also performed to determine whether the ratio of produced to target time changed significantly according to the target time. Here, the dependent variable was the ratio of produced time to target time for each participant for each target time, and the independent variable was the target time.

#### Other statistical analyses

Power calculations were performed using GWASpower/QT [64] (available at http://igm.cumc.columbia.edu). A one-sample mean comparison test was performed in STATA to determine whether the distribution of the ratio of time produced to target time for each target time differed from 1 (meaning that people were statistically significantly more likely to underproduce).

### Genetic Analyses

A genome-wide association study (GWAS) was performed on 148 participants who had Illumina Humanexome chip data available. Of these 148, most were also genotyped with the Infinium HumanCore GWAS chip (n = 113), and others were genotyped with either the Human610-Quad BeadChip (n = 10) or HumanHap550 (n = 14). Eleven of these 148 samples did not have GWAS genotypes. Variants from any of these chips were included in the analysis provided they passed QC and met the below inclusion criteria.

Our single variant analysis restricted to variants genotyped in at least 50% of these participants with a minor allele frequency (MAF) of at least 0.01. A linear regression was used in plink [65] for the counting (n = 146), timing (n = 115) and duration discrimination phenotypes (n = 145). We analyzed only those of European ancestry. Two EIGENSTRAT axes and PC1 were used as covariates in each analysis, with sex also used as a covariate in the timing analyses. A total of 274,814 variants were analyzed in this GWAS. Correction for multiple tests therefore required a p-value of 1.8x10^-7^ to declare significance.

To assess the effects of the low frequency variants genotyped with the exome chip, we used a gene-based collapsing analysis as previously described [66]. Briefly, we summarized for each participant whether there existed a ‘qualifying’ variant in each gene, where qualifying was defined as an exonic variant with MAF < 0.01. Linear regression analysis was then performed with two EIGENSTRAT axes and PC1 as covariates. This allows the identification of genes where qualifying variants are enriched in individuals toward one extreme or the other of each trait.

To increase power, a focused analysis of the GWAS and exome chip data was also performed on genes that have been previously hypothesized to have an influence on time perception: COMT, DRD2, MAOA, ANKK1, SLC6A3, SLC6A4, PER1, PER2, CRY1, CRY2, SIK1 and HTR2A [16, 43, 46, 47, 67]. A 10 kilobase flanking region around these genes was considered, resulting in 107 variants included, requiring a p value of 4.67 x10^-4^ to reach significance. Note that repeat variants within these genes are not assessed with chip genotyping.

## Results

### Time Production: Counting

**Fig 2:**
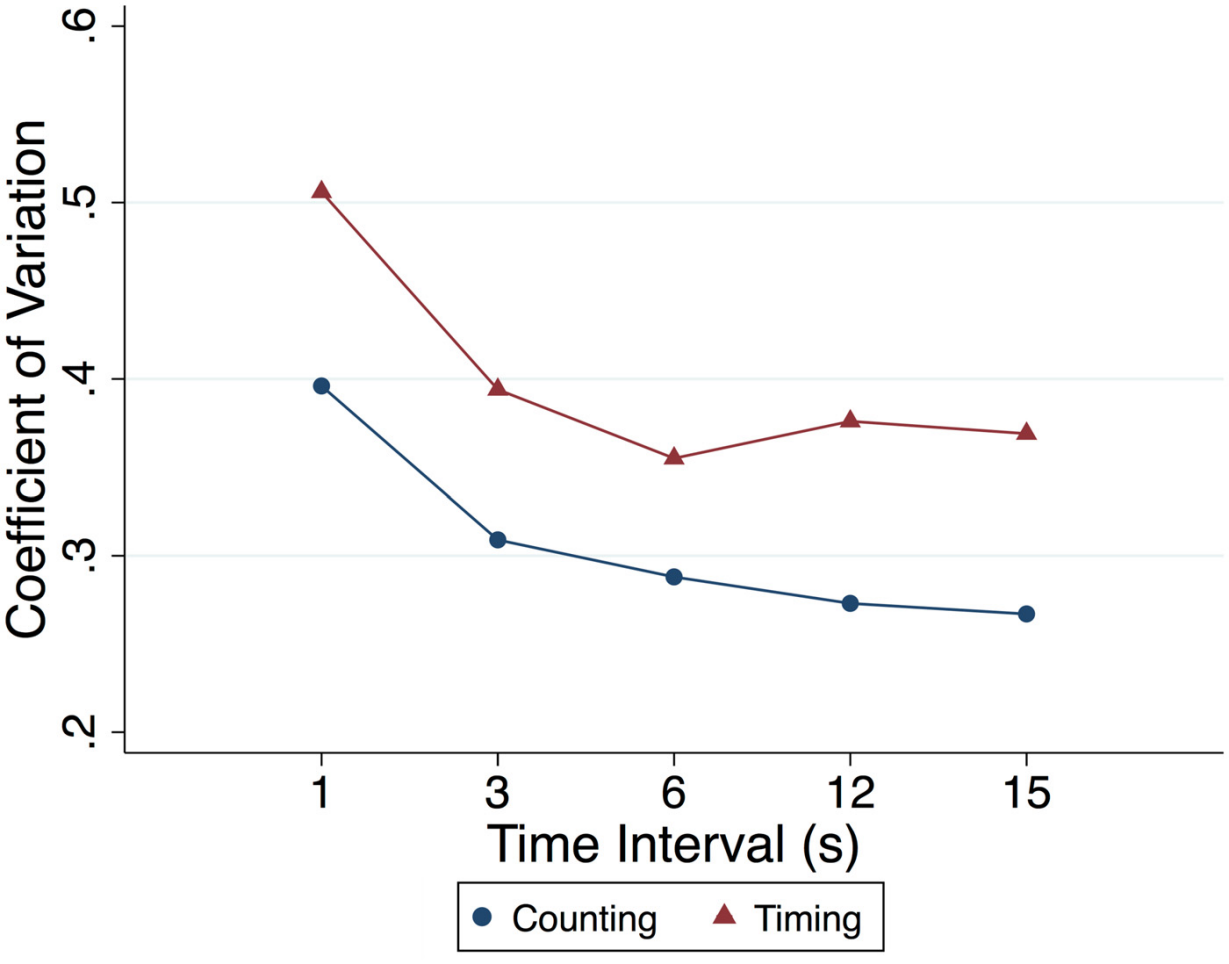
Group coefficient of variation (CV). Group CV for the counting and timing conditions at each requested interval (1, 3, 6, 12, 15-second) of the time production task.

Participants were requested to produce time intervals of 1, 3, 6, 12, and 15 sec by using a counting/sub-division method of their choice. Group means were underestimated at each interval (p < 0.0001), and there was a significant trend for underproduction to decrease as the interval lengthened (p < 0.001; ratios of 0.73, 0.76, 0.81, 0.85, and 0.88 respectively for 1, 3, 6, 12 and 15 sec). The log-transformed ratio of produced time to requested time was well correlated (r > 0.75) across the range of 3 to 15 sec within participants as illustrated in Fig 1. The group CV was 0.40 at the 1-sec trial and decreased slightly in a narrow range throughout the 3-, 6-, 12-, and 15-sec production trials (range: 0.31–0.27; Fig 2).

### Time Production: Timing

During the timing condition, 491 participants reported being able to refrain from using a counting strategy. We did not find a significant difference in the time production phenotype based on whether or not the participant was able to refrain from counting (*p* = 0.95). Nonetheless, we restricted analyses of the timing condition to those participants who claimed to be able to resist counting.

While eliminating the use of counting strategies, participants again underproduced the requested intervals (*p* < 0.0001; ratios of 0.67. 0.67, 0.65, 0.64, and 0.72 respectively for 1, 3, 6, 12, and 15 sec). The log-transformed ratio of produced time to requested time was well correlated across the range of 3 to 15 sec within participants to a lesser degree (r > 0.58) than the counting condition trials as illustrated in Fig 1. The group CV was 0.51 at the 1-sec trial and remained relatively constant during the 3- to 15-sec trials (range: 0.36–0.39; Fig 2).

For all subsequent analyses, we restricted to the 3–15 sec range to reflect the greater reliability and correlation amongst these intervals in both conditions, as illustrated in Fig 1.

**Fig 3:**
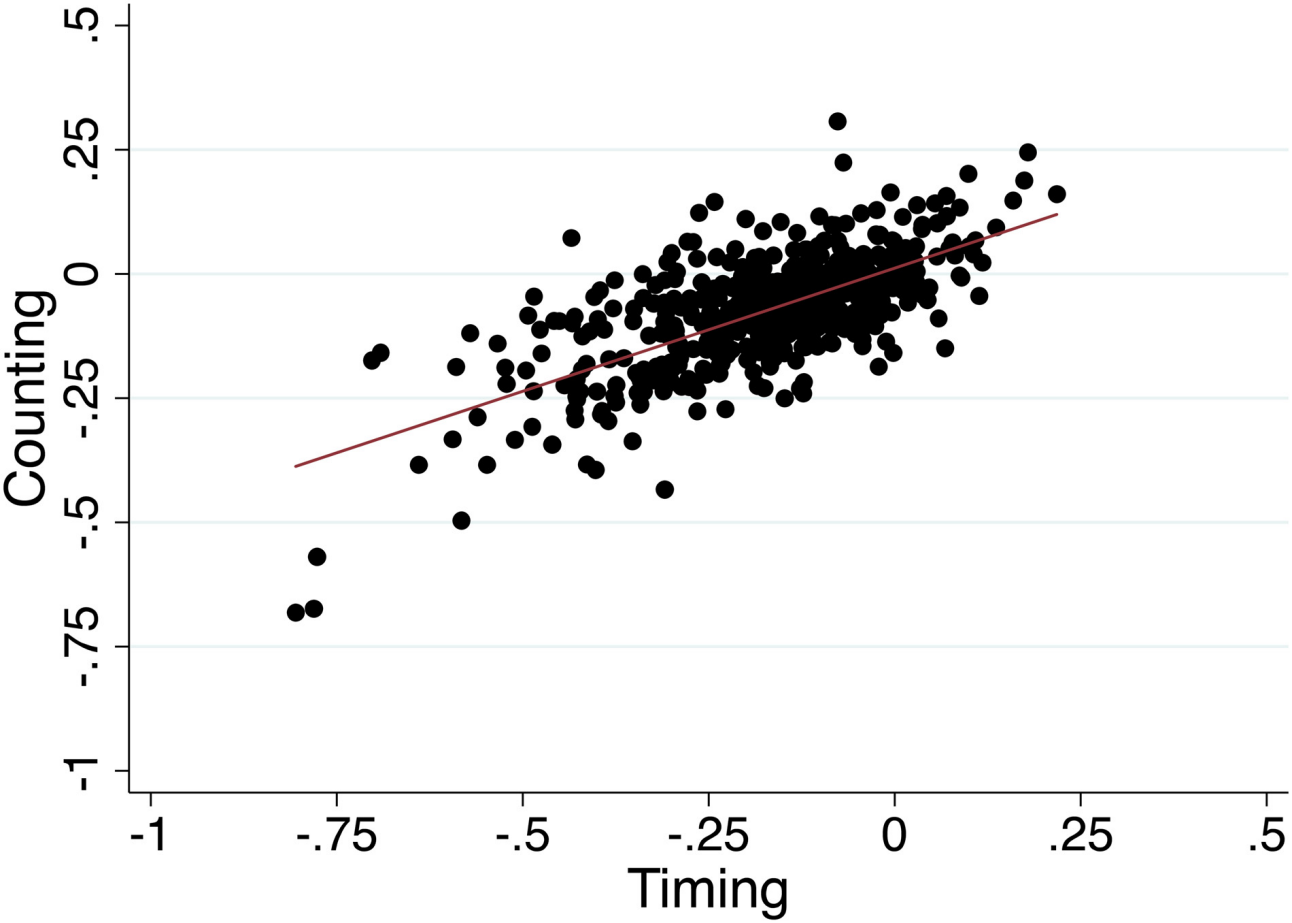
Counting vs. timing accuracy. Correlation between the timing and counting conditions of the time production task (Pearson’s r = 0.68, *p* < 0.001). Values are plotted as the median of the log-transformed ratio of produced time to requested time at the 3, 6, 12, and 15-second time intervals.

Performance on the *timing* and *counting* conditions of the production task was well correlated (*p* < .001, r = 0.68; Fig 3). Approximately 45.8% of the variation in one testing condition could be explained by performance on the other condition.

### Duration Discrimination

Participants required a mean of 42.7 msec (SD = 13.8) to discriminate temporally similar tones. Performance on the duration-discrimination task was weakly, but significantly associated with performance on the *timing* (r = -0.14; p = 0.003) and *counting* (r = −0.16; p < 0.001) conditions of the production task. CV values for each participant were calculated using the three independent thresholds (mean = 0.23, SD = 0.14).

### Repeated Testing Sessions for Time Production and Duration Discrimination

A total of 46 participants completed the time production and duration discrimination tasks twice, allowing us to investigate the stability of timing ability over relatively long periods of time. After exclusions, a total population of 44 for counting production, 45 for timing production, and 43 for duration discrimination were included in repeat analyses.

**Fig 4:**
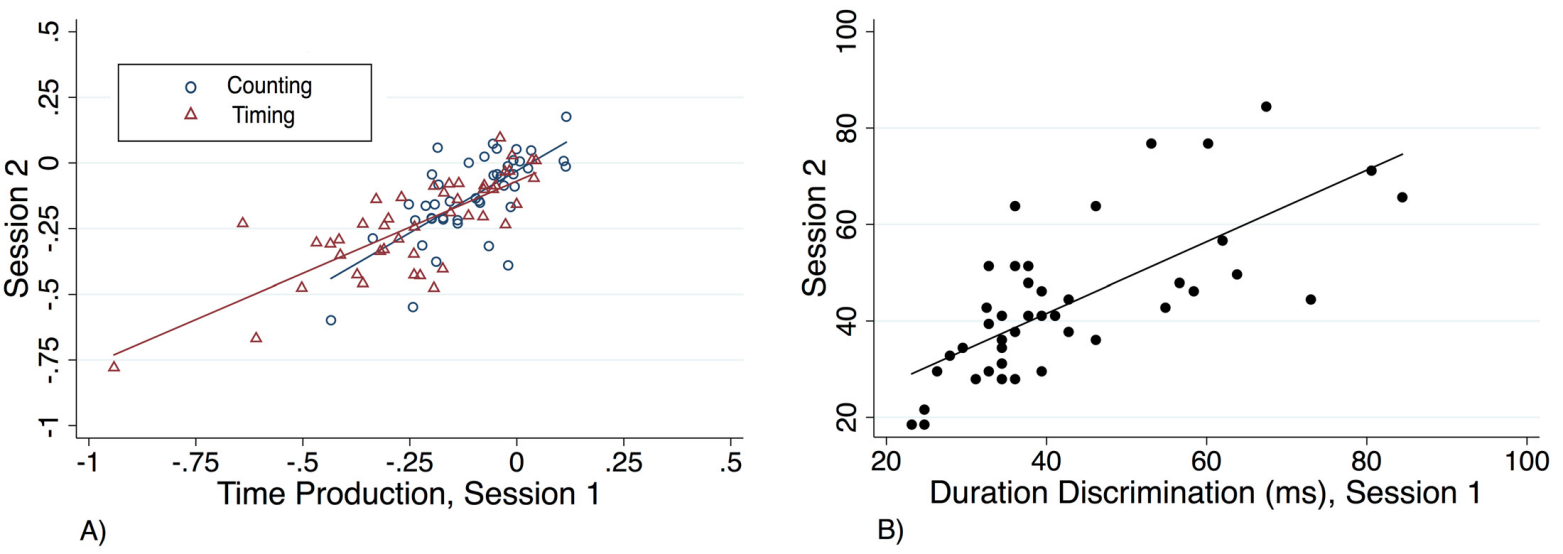
Repeat testing sessions. Performance during session one and session two for a) both counting (Pearson’s r = 0.70; *p* < 0.001) and timing (r = 0.78; *p* < 0.001) conditions of the time production task, as well as the b) duration discrimination (r = 0.71; *p* < 0.001) task.

Time production performance on the initial testing session was strongly correlated with the second session and explained 47.1% of the variation in the *counting* condition and 61.2% of the variation in the *timing* condition of the production task (*p* < .001). We obtained a comparably strong Pearson’s r and Spearman’s rho for both conditions between the separate occasions (counting: r = 0.69, rho = 0.64; timing: r = 0.78, rho = 0.77; Fig 4a).

Duration discrimination performance was also well correlated across testing sessions (p < 0.001), and performance on the first session explained 50.9% of the variation in the second session. Pearson and Spearman statistics were similar to time production repeats (r = 0.71, rho = 0.73; Fig 4b).

### Multivariate stepwise regressions: Time production and duration discrimination

We systematically assessed the contribution of age, ethnicity, sex, intelligence, level of education, circadian preference, extraversion, neuroticism, musical training and ADHD diagnosis to individual variation in performance on time production and duration discrimination tasks (Table 2).

Intelligence and ethnicity met inclusion thresholds (*p* < 0.01) in stepwise models for all three phenotypes: *timing* production, *counting* production, and duration discrimination. Unique to the timing model, sex also met inclusionary criteria due to females underproducing target durations. In addition, African Americans and, to a lesser extent, Hispanics, significantly underproduced target durations during the production task (for Hispanics, only counting was different) and had significantly larger duration discrimination thresholds compared to other ethnic groups.

Taken together, intelligence and ethnicity explained 12.0%, 15.7%, and 8.9% of the variation in the duration discrimination, *counting* production, and *timing* production tasks, respectively. The inclusion of sex in the *timing* production model increased the variation explained to 12.5%. When restricting stepwise regression analyses to a student population under the age of 25, ethnicity no longer met inclusionary criteria in any of the models.

### Cognitive correlates

Each of the eleven cognitive measures of our cognitive battery were strongly (*p* < 0.001) associated with performance on *counting* production and duration discrimination tasks (Table 3). The *timing* production task had the lowest correlation with these different cognitive tests, but all associations still had *p* < 0.01 except for Digit Span Forward (p = 0.012), COWA (p = 0.064), and Animals (0.275) subtasks. The *counting* production and duration discrimination tasks showed the highest correlations with tests assessing executive function, processing speed, and verbal episodic memory, without a strong signal for performance reflecting only one aspect of cognition. The strongest correlation was found between each of these variables and overall performance on the cognitive battery, our proxy for general intelligence. Indeed, principal component analysis of the standard cognitive tests and the timing phenotypes presented here found that the timing phenotypes loaded onto the first principal component at levels similar to that of the digit span forward task, which is the cognitive test with the lowest loading onto this component.

**Table 3:** Cognitive correlates with timing accuracy and precision.

Test	PCA loading for general intelligence	Cognitive Area	Production [Counting]	Production [Timing]	Duration Discrimination
TrailsB [68]	-0.36	Attention, Processing Speed, Executive Control	-0.24	-0.17	0.26
Symbol Search [69]	0.34	Processing Speed, Executive Control	0.23	0.18	-0.23
Delayed Story Recall [70]	0.33	Verbal Episodic Memory	0.24	0.13	-0.17
Stroop Color-Word [71]	0.33	Attention, Executive Control	0.20	0.17	-0.28
Immediate Story Recall [70]	0.32	Verbal Episodic Memory	0.20	0.15	-0.16
Animals [72]	0.32	Semantic Fluency	0.14	0.05*	-0.15
TrailsA [68]	-0.30	Attention, Processing Speed	-0.23	-0.16	0.15
Digit Symbol [69]	0.29	Processing Speed, Working Memory, Executive Control	0.21	0.17	-0.16
COWA [73]	0.26	Verbal Fluency, Executive Control	0.18	0.08*	-0.19
Digit Span Backward [69]	0.25	Working Memory	0.18	0.15	-0.16
Digit Span Forward [69]	0.19	Working Memory	0.15	0.11*	-0.11

Correlations between subtests of the cognitive battery and timing performance are presented as Pearson’s r. All *p* < 0.01 except those with *.

### Genetic associations

After correcting for multiple tests, we identified no variants or genes with statistically significant associations with these traits. Our genome-wide association study had 80% power to identify a common variant explaining at least 19% of the variation in this trait, and our gene-based collapsing analysis of low-frequency coding variants had 80% power to identify associations explaining at least 17% of the variation. When restricting to 12 candidate genes, we still found no significant associations despite having 80% power to identify associations explaining at least 8–11% of the variation in these traits.

## Discussion

We have assessed a large population of healthy volunteers for their performance on time production and duration discrimination tasks. Performance was analyzed in the context of numerous other variables thought to influence timing and time perception in the seconds-to-minutes range, including age, sex, depression, education, and cognitive performance. Due to the vast majority of the variation in the employed timing tasks remaining unexplained, we performed preliminary genetic analyses to search for biological correlates that explain the range of individual variation in timing and time perception [27].

Our results confirm the stability of an individual’s temporal accuracy and precision across supra-second intervals (ranging from 3 to 15 sec) and across multiple sessions separated by several months [74–76]. We confirm that female participants are more likely to underestimate in a prospective time production task when an explicit counting strategy is not employed. A previous review highlighted that a lack of association between time perception and sex was often reported based on a single participant response [27, 77]. In studies that use multiple trials to generate a time production phenotype, sex effects on clock speed tend to be small, but reliable [78], as we find here. However, it should be noted that the effect of sex was not significant during the *counting* condition of our time production task.

There remains discordance in the literature on the effect of aging and clock speed. Increasing age may lead to a decrease in the accuracy of time estimation and production, though the direction of this effect remains unclear [79]. The perception that events in the external world are occurring faster is commonly reported, as well as a slower preferred motor tempo [29, 80, 81]. Both of these effects have been attributed to a slower speed of the internal clock, possibly due to the use of longer internal refrants in older participants [81–84]. Moreover, there is evidence that age-related cognitive declines may be at the root of inconsistent findings [26, 80]. In our study, we do not find a significant effect of age when taking intelligence into account. However, prior to taking intelligence into account, there was a strong trend for older individuals to underproduce requested intervals and to have larger duration discrimination thresholds. A recent study also reported that age did not affect the production of time intervals under 20 sec [27]. Thus, our data support the theory that the contested aging effect seen in the literature is due to a failure to take into account age-related cognitive performance [26, 85]. Specifically, it was previously identified that processing speed in older participants was at the root of age-related differences [80]. We likewise find an association between musical training and clock speed that is accounted for by taking intelligence into account.

Due to a strong association of intelligence with both time production and duration discrimination, we further examined specific aspects of cognition. Our intelligence battery captures aspects of episodic memory, attention, processing speed, working memory, and executive function, and uses task performance to generate a single measure that is a proxy for general intelligence. We find significant associations (*p* < 0.001) for all eleven tasks (Table 3) with both *counting* production and duration discrimination. Better performance corresponded to better accuracy on the time production task and smaller discrimination thresholds. Overall, general intelligence was the best predictor of performance for all of our timing measures. Moreover, our findings indicate that the accuracy and precision of interval timing is a primary dimension of general intelligence—in agreement with previous work showing that intelligence and variability in timing tasks share common neural substrates in sensory cortices including prefrontal white matter [86–89]. A degree of caution is advisable, however, when considering our measures for the accuracy and precision of timing because participants were evaluated using a duration-discrimination task in the sub-second range (precision) and a time-production task in the supra-second range (accuracy). Although differences in these measures have been observed as a function of time scale, there is considerable evidence supporting the application of a common metric across different timing tasks.

The current literature is still developing as to what role working memory and processing speed play in timing and time perception. The temporal resolution power (TRP) hypothesis posits that working memory capacity largely mediates the connection between temporal information processing and psychometric intelligence [90]. Multiple studies have demonstrated and replicated a significant correlation between temporal processing, (e.g., duration discrimination, temporal-order judgment, and temporal generalization tasks) and psychometric intelligence [91–93]. This body of work consistently demonstrates a large amount of variation (>30%) in intelligence accounted for by temporal processing, suggesting that TRP may reflect a facet of general intelligence [92]. We do not find working memory tasks to be the most correlated with our timing tasks. Instead, we find that measures of executive function play a larger role, and that general intelligence shows the best association with timing tasks. On the other hand, there has also been evidence of a double dissociation between time reproduction, dependent on working memory, and time production, dependent on clock speed and correlated with preferred motor tempo [29]. Our work adds to several other studies that demonstrate interplay between general intelligence, working memory, and timing abilities in healthy adults [83, 94].

We also demonstrate, for the first time, a significant association between ethnicity and sense of time. Specifically, we identify that African Americans and Hispanics underestimate on time production tasks (only *counting* production for Hispanics) and have higher duration discrimination thresholds as compared to European Americans, even when accounting for education, age and general cognitive function. However, when restricting to a college student population, the difference in ancestry was no longer significant. This difference by ancestry could be due to underlying genetic variation playing a role in this trait that differs among these populations, or to socioeconomic and societal differences among these groups that we are unable to account for in the present study.

While our study is one of the largest reported in terms of population size, our timing phenotypes were generated based on single time production trials at each of four requested time intervals ranging from 3 to 15 sec. This design means that we are unable to fully characterize the extent of variability in a series of measures within a single participant and to determine whether the scalar property of interval timing correlates with any of our other cognitive or genetic measures [18]. In addition, all of our target durations were underproduced, in both the counting and timing conditions of the production task. This result is not particularly surprising as feedback was not provided to the participants and they were unable to adapt their performance accordingly [59].

Despite these limitations, our successful characterization of a large and healthy cohort on time production and duration discrimination tasks helps to lay the foundation for future genetic analyses. Our data show that the vast majority of the variability in time sense is unexplained by the comprehensive factors we assessed, consistent with a genetic component to the perception and estimation of time. Our preliminary genetic studies, which admittedly have very small sample sizes, are nonetheless sufficiently powered to rule out the possibility that a single common variant or groups of low-frequency coding variants within a single gene explain more than ~18% of the variation in this trait. These results are unsurprising given the findings for genome-wide studies of most complex traits, but they do provide an important context in which to pursue additional genetic studies. Our results additionally show no support for a role of previously assessed candidate genes in these traits, e.g., COMT, DRD2, MAOA, ANKK1, SLC6A3, SLC6A4, and HTR2A [43, 46, 47]. These preliminary genetic data combined with the stability of these traits argue for additional studies in this area that utilize genome-wide data on a large number of individuals, including the investigation of pharmacogenetic traits related to timing and time perception [10, 33, 95, 96].

## Supporting Information

S1 File Phenotypes and Covariates. Phenotypic and covariate information for the samples included in this study.
(XLSX)

S2 File Collapsing Analyses. Results from the gene-based collapsing analyses included in this study.
(XLSX)

S3 File GWAS Analyses. Results from the GWAS analyses included in this study.
(ZIP)
